# Maturation of the cytochrome *cd*_1_ nitrite reductase NirS from *Pseudomonas aeruginosa* requires transient interactions between the three proteins NirS, NirN and NirF

**DOI:** 10.1042/BSR20130043

**Published:** 2013-06-27

**Authors:** Tristan Nicke, Tobias Schnitzer, Karin Münch, Julia Adamczack, Kristin Haufschildt, Sabine Buchmeier, Martin Kucklick, Undine Felgenträger, Lothar Jänsch, Katharina Riedel, Gunhild Layer

**Affiliations:** *Institute of Microbiology, Technische Universität Braunschweig, Spielmannstr. 7, 38106 Braunschweig, Germany; †Institute of Physical and Theoretical Chemistry, Technische Universität Braunschweig, Hans-Sommer-Street 10, 38106 Braunschweig, Germany; ‡Department of Cellular Proteome Research, Helmholtz Centre for Infection Research, Inhoffenstr. 7, 38124 Braunschweig, Germany; §Institute of Microbiology, Ernst-Moritz-Arndt University of Greifswald, Friedrich-Ludwig-Jahn-Street 15, 17489 Greifswald, Germany

**Keywords:** co-immunoprecipitation, cytochrome *cd*_1_, denitrification, haem *d*_1_, tetrapyrroles, CV, column volume, N_2_O, nitrous oxide, rbs, ribosome binding site, WT, wild-type.

## Abstract

The periplasmic cytochrome *cd*_1_ nitrite reductase NirS occurring in denitrifying bacteria such as the human pathogen *Pseudomonas aeruginosa* contains the essential tetrapyrrole cofactors haem *c* and haem *d*_1_. Whereas the haem *c* is incorporated into NirS by the cytochrome *c* maturation system I, nothing is known about the insertion of the haem *d*_1_ into NirS. Here, we show by co-immunoprecipitation that NirS interacts with the potential haem *d*_1_ insertion protein NirN *in vivo*. This NirS–NirN interaction is dependent on the presence of the putative haem *d*_1_ biosynthesis enzyme NirF. Further, we show by affinity co-purification that NirS also directly interacts with NirF. Additionally, NirF is shown to be a membrane anchored lipoprotein in *P. aeruginosa*. Finally, the analysis by UV–visible absorption spectroscopy of the periplasmic protein fractions prepared from the *P. aeruginosa* WT (wild-type) and a *P. aeruginosa* Δ*nirN* mutant shows that the cofactor content of NirS is altered in the absence of NirN. Based on our results, we propose a potential model for the maturation of NirS in which the three proteins NirS, NirN and NirF form a transient, membrane-associated complex in order to achieve the last step of haem *d*_1_ biosynthesis and insertion of the cofactor into NirS.

## INTRODUCTION

The opportunistic human pathogen *Pseudomonas aeruginosa* performs denitrification for energy generation under micro-aerobic and anaerobic growth conditions in the presence of nitrate [1]. In *P. aeruginosa*, the second step of denitrification is catalysed by the periplasmic cytochrome *cd*_1_ nitrite reductase NirS [2]. In addition to its important function during denitrification, NirS also seems to contribute to the virulence of *P. aeruginosa* by producing the signal molecule NO [3]. NirS is a homodimeric protein in which each monomer consists of two distinct domains. The N-terminal domain resembles small *c*-type cytochromes and contains a covalently bound haem *c*. The C-terminal domain possesses an eight-bladed β-propeller fold and contains the catalytically essential haem *d*_1_, which is non-covalently bound in the active site of the enzyme [4].

**Figure 1:**
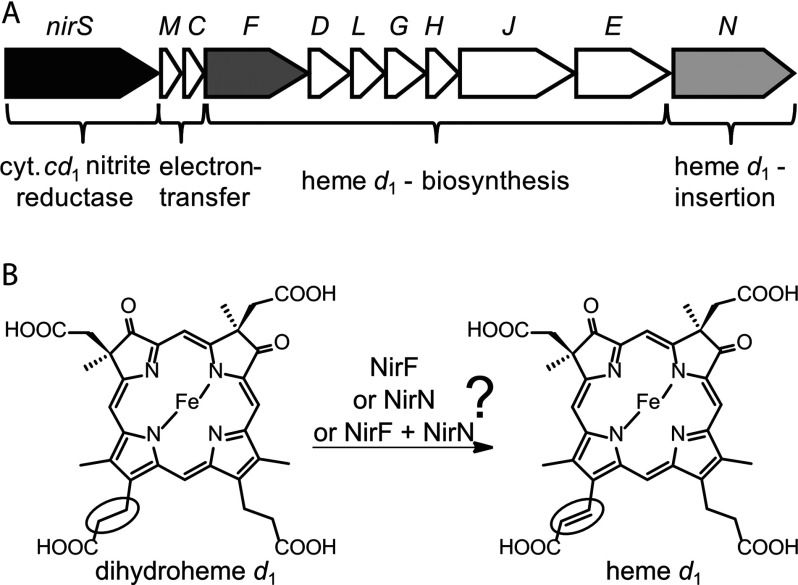
Genes and proteins required for haem *d*_1_ biosynthesis and insertion (**A**) The *nir* operon of *P. aeruginosa*. The *nirS* gene encodes the cytochrome *cd*_1_ nitrite reductase NirS. The genes *nirM* and *nirC* encode two small *c*-type cytochromes. The proteins encoded by the genes *nirFDLGHJE* are involved in haem *d*_1_ biosynthesis. NirN is potentially required for haem *d*_1_ insertion into NirS. (**B**) The last step of haem *d*_1_ biosynthesis. It was proposed that the last step of haem *d*_1_ biosynthesis is the dehydrogenation of one of the propionate side chains of dihydrohaem *d*_1_ to the corresponding acrylate side chain of haem *d*_1_ (highlighted by a circle) [16]. This reaction might be catalysed by NirF or NirN or both NirF and NirN.

Whereas NirS itself is a well-characterized enzyme [5–8], there is less knowledge about the maturation that is, cofactor insertion, of this protein. The cofactor-free apo NirS is transported from the cytoplasm to the periplasm *via* the Sec pathway [9]. In the periplasm, the haem *c* is incorporated into NirS by the cytochrome *c* maturation system I [10]. However, so far nothing is known about the insertion of the haem *d*_1_ into NirS. Some of the proteins that are potentially involved in haem *d*_1_ biosynthesis and insertion into NirS are encoded by the various *nir* genes localized in the *nir*-operon of *P. aeruginosa* (Figure 1A) [11]. The *nirFDLGHJE* genes encode proteins required for haem *d*_1_ biosynthesis as demonstrated by mutational studies and biochemical characterization [11–16]. Most steps of haem *d*_1_ biosynthesis are believed to take place in the bacterial cytoplasm with the exception of the last step, which was suggested to take place in the periplasm [17]. It was speculated that the last step of haem *d*_1_ biosynthesis could be the dehydrogenation of one of the propionate side chains to the corresponding acrylate side chain of haem *d*_1_ (Figure 1B) [16]. Further, it was proposed that NirF could catalyse this last biosynthesis step based on the observation that NirF from *Paracoccus pantotrophus* is a soluble, periplasmic protein able to bind haem *d*_1_ [17]. In contrast, *P. aeruginosa* NirF was annotated as a cytoplasmic protein in the databases and was only recently predicted to be a periplasmic lipoprotein in a bioinformatics study [18].

Assuming that NirF indeed catalyses the last step of haem *d*_1_ biosynthesis, the cofactor has to be transferred from NirF to NirS. It was proposed that the NirN protein might be involved in this haem *d*_1_ transfer and insertion process [15]. *P. aeruginosa* NirN shares about 24% amino acid sequence identity with NirS from this species. It was shown that NirN is a soluble, periplasmic cytochrome *c* [19]. Further, *P. pantotrophus* NirN was able to bind haem *d*_1_ and to transfer the cofactor to NirS *in vitro* [15]. However, the exact role of NirN *in vivo* remained unclear, since the deletion of the *nirN* gene in *P. aeruginosa* and *P. pantotrophus* did not completely abolish the formation of a catalytically active NirS, but only resulted in less NirS activity in cell-free extracts prepared from the Δ*nirN* mutant strain than in cell-free extracts prepared from the WT (wild-type) strain [11,15,19]. This observation allows for the proposal of two potential roles for NirN. Firstly, NirN might indeed be involved in the insertion of haem *d*_1_ into NirS, but is not absolutely essential for this process. Secondly, NirN might be involved in both the last step of haem *d*_1_ biosynthesis (possibly together with NirF) and the subsequent insertion of the cofactor into NirS. If the second scenario was true, the haem *d*_1_ precursor carrying a propionate side chain instead of the acrylate side chain (dihydrohaem *d*_1_) could still be incorporated into NirS leading to a ‘semi-functional’ NirS. Indeed, it was shown *in vitro* that NirS from *Pseudomonas stutzeri* still exhibited about 50% NirS activity when its native haem *d*_1_ was replaced by the synthetic haem *d*_1_ precursor lacking the double bond of the acrylate side chain [20].

This study was conducted in order to obtain more insights into the *in vivo* function of the potential haem *d*_1_ insertion protein NirN from *P. aeruginosa*. We could show that NirN interacts with NirS *in vivo*, and we established that NirF from *P. aeruginosa* is a membrane attached lipoprotein, which also directly interacts with NirS. Further, we examined the effects of the *nirN* knock out on the cofactor content of NirS and observed that in the absence of NirN the cofactor content of NirS is altered. Based on our results we propose a new working model in which the formation of a NirF-NirN-NirS complex is required for the maturation of NirS.

## MATERIALS AND METHODS

### Chemicals and polyclonal antibodies

All chemicals and reagents were purchased from Sigma-Aldrich, Merck or Thermo Fisher Scientific Inc. Restriction enzymes were obtained from New England Biolabs. QIAquick PCR-Purification and gel extraction Kits were purchased from Qiagen GmbH. The QuikChange Kit was obtained from Agilent Technologies. Q-Sepharose Fast Flow and SP-Sepharose Fast Flow were obtained from GE Healthcare, protein G-agarose and protein A-agarose was purchased from GenScript USA Inc., Strep-Tactin-HC-resin, Strep-Tactin-AP conjugate, Desthiobiotin and Avidin were obtained from IBA GmbH. All primers and polyclonal antibodies (rabbit) were purchased from Metabion International AG. Secondary antibodies against rabbit IgG AP-conjugate, mouse IgG (light chain) AP-conjugate and mouse IgM AP-conjugate were purchased from Dianova GmbH. Polyvinylidene fluoride membrane was obtained from Merck Millipore. SYPRO® Ruby was obtained from Life Technologies GmbH.

### Strains and plasmids

*Escherichia coli* DH10B was used as the host for cloning. For protein production of semi-apo-NirS and NirN, *E. coli* BL21 (DE3) was transformed with plasmid pEC86 (provided by Dr Linda Thöny-Meyer [21]) and either pET22b*nirS* or pET22b*nirN*. For growth experiments, complementation studies and the screenings for monoclonal antibodies *P. aeruginosa* PAO1 WT and *P. aeruginosa* PAO1 strains RM488 (*nirS*::*tet*), RM361 (*nirN*::*tet*) and RM301 (*nirF*::*tet*) were used (provided by Dr Hiroyuki Arai [11]). The transfer of plasmids into *P. aeruginosa* PAO1 strains was done by diparental mating using *E. coli* ST 18 as described previously [22]. *P. stutzeri* ZoBell MK202 pUCP-Nir (provided by Dr Francesca Cutruzzola [23]) was used to produce holo-NirS.

### Construction of vectors

The *nirS* gene was amplified by PCR from *P. aeruginosa* PAO1 genomic DNA with primers 1 and 2 (all primers used in this study are listed in Supplementary Table S1 at http://www.bioscirep.org/bsr/033/bsr033e048add.htm). The resulting DNA fragment was digested with NcoI and HindIII and ligated into the vector pET22b(+) to generate the plasmid pET22b*nirS* coding for a PelB-NirS fusion protein. The *nirN* gene was amplified by PCR using *P. aeruginosa* PAO1 genomic DNA and primers 3 and 4, digested with NcoI and BamHI and ligated into pET22b(+) to generate pET22b*nirN* encoding a PelB–NirN fusion protein. For the construction of pUCP20T*nirFOneSTrEP* the *nirF* gene including its rbs (ribosome binding site) was amplified from *P. aeruginosa* PAO genomic DNA by PCR with primers 5 and 6, digested with BamHI and SpeI and ligated into pJ201-*OneSTrEP* (provided by the group of Dr Ralf-Rainer Mendel, Institute of Plant Biology, TU Braunschweig). The generated *rbs-nirF-OneSTrEP* fragment was amplified by PCR with primers 5 and 7 to incorporate an additional SphI restriction site downstream of the *nirF-OneSTrEP* stop codon for ligation into the broad host range vector pUCP20T using the BamHI and SphI restriction sites. For the construction of the vector pUCP20T*nirF*, a stop codon was inserted downstream of the *nirF* coding sequence by site-directed mutagenesis using the QuikChange Kit with primers 8 and 9 and pUCP20T*nirFOneSTrEP* as template DNA. The construct pUCP20T*nirFStrepII* was generated by site directed mutagenesis using the QuikChange Kit with primers 10 and 11 and pUCP20T*nirFOneSTrEP* as template DNA to shorten the OneSTrEP-tag by introduction of a stop codon downstream of the first Strep-Tag coding sequence. The nucleotide sequences of cloned constructs were verified by DNA-sequencing (GATC Biotech AG).

### Growth conditions

*E. coli* BL21 (DE3) carrying the plasmids pEC86 and pET22b*nirS* or pET22b*nirN* for the production of semi-apo NirS (containing the covalently bound haem *c*, but no haem *d*_1_) and haem *c* containing NirN, respectively, were grown according to Studier [24] in self-inducing medium ZYM5052 containing 100 μg/ml ampicillin and 34 μg/ml chloramphenicol for 4 h at 37°C and subsequently for 26 h at 25°C. *P. aeruginosa* PAO1 WT and mutant strains were grown anaerobically as reported before [14]. *P. stutzeri* ZoBell MK202 pUCP-Nir was grown as described previously [23].

### Purification of proteins

The purification of recombinant *P. aeruginosa* semi-apo NirS, holo NirS and NirN was performed according to Parr *et al.* [25].

### Monoclonal antibodies

The monoclonal antibodies were generated by immunizing mice with purified semi-apo-NirS or NirN, respectively, according to a standard immunization protocol. After hybridization and cloning, antibody producing hybridoma cells were screened by ELISA for their binding ability to purified antigen. Isotype analysis of the strongly reactive clones 1A11, 4A9, 2B8, 6E4 and 1G12 against NirS revealed an IgG1 subtype. The clones 1B12, 6C5, 2D6 and 3G9 against NirS revealed an IgG2a subtype. Against NirN one strongly reactive clone (2C11) was obtained producing IgM subtype antibodies. All monoclonal antibodies were screened against *P. aeruginosa* PAO1 cell-free extracts, prepared from the WT strain and the RM488 and RM361 mutant strains without showing significant cross selectivity against *P. aeruginosa* proteins other than the antigen. Antibody-containing supernatants were gained according to standard protocols. The αNirS 1A11 from growth media was used for Western blot staining of NirS. For immunoaffinity chromatography of NirS a mix of all generated monoclonal antibodies against NirS was used. The IgM-type antibody 2C11 (αNirN) was used from growth media for Western blot staining of NirN.

### *In vivo* protein cross-linking

The *in vivo* protein cross-linking was performed as described previously [26] with minor modifications (for details see Supplementary Material at http://www.bioscirep.org/bsr/033/bsr033e048add.htm).

### Co-immunoprecipitation

For the co-immunoprecipitation of native NirN and NirS from *P. aeruginosa* cell-free extracts, anaerobically grown *P. aeruginosa* cells were harvested in the late exponential phase after *in vivo* protein cross-linking. The cell pellet (0.5 g) was resuspended in 5 ml of a previously described lysis buffer [27] supplemented with 0.5% (w/v) sodium-deoxycholate and 0.5% (w/v) *n*-octylglucoside, but omitting DTT (dithiothreitol). The cells were disrupted by FastPrep (MP Biomedicals) by the addition of 250 mg glass beads to 750 μl cell suspension and disruption at 4°C for 3×45 s shaking with 5.5 m/s. Cell debris were removed by centrifugation (20000 ***g***) for 30 min at 4°C. 750 μl of the resulting supernatant were incubated with 2 μg of αNirS or αNirN polyclonal rabbit antibodies for 90 min at 4°C. Further incubation with 80 μl of protein-A-agarose in lysis buffer (1:1 slurry) was carried out by gentle shaking for 90 min at 4°C. The immunoabsorbant was separated from the supernatant by centrifugation (16100 ***g***) for 1 min at room temperature and washed afterwards three times with 650 μl washing buffer containing 50 mM Tris/HCl pH 8.0, 150 mM NaCl, 0.1% (v/v) Nonidet P40, 0.1% (w/v) sodium-deoxycholate, 0.1% (w/v) *n*-octylglucoside and 1 mM EDTA. The samples were treated with 35 μl 2×SDS/PAGE sample buffer supplemented with 2-mercaptoethanol and heated for 10 min at 95°C. After SDS/PAGE and Western blotting, the proteins were visualized as described in the Supplementary Material (at http://www.bioscirep.org/bsr/033/bsr033e048add.htm).

### Affinity co-purification with Strep-Tactin sepharose

Cell-free extracts of *P. aeruginosa* PAO1 RM301 carrying pUCP20T*nirFOneSTrEP* or pUCP20T (control) were prepared as described above for the co-immunoprecipitation procedure. 500 μl of the cell free extract were incubated with 200 μl of Strep-Tactin-HC-resin in lysis buffer (1:1 slurry). The incubation of the resin, the washing steps and sample preparation were performed as described above for co-immunoprecipitation.

### Preparation of membrane fractions from *P. aeruginosa*

*P. aeruginosa* cells were resuspended in 50 mM Tris/HCl, pH 8.0, containing 15 mM EDTA and disrupted by passage through a French-Pressure cell at 1000 psi (1 psi = 6.9 kPa). To remove cell wall fragments the obtained cell-free extract was centrifuged for 20 min at 20000 ***g*** and 4°C. The resulting supernatant was again centrifuged for 1 h at 100000 ***g*** to generate a membrane pellet, which was washed five times with resuspension buffer. Afterwards, the membrane proteins were solubilized in lysis buffer. Solubilized proteins were then separated from the insoluble membrane fraction by centrifugation for 1 h at 100000 ***g*** and 4°C.

### Preparation of the inner and outer membrane fractions from *P. aeruginosa*

For the separation of the inner and outer membrane fractions of *P. aeruginosa*, the cells were resuspended in membrane separation buffer (100 mM potassium acetate, 5 mM magnesium acetate, 50 mM HEPES, 0.05% (v/v) 2-mercaptoethanol, Complete™ protease inhibitor cocktail EDTA free (Roche) at pH 7.5) and disrupted with a French-Press. Undisrupted cells were pelleted by centrifugation at 10000 ***g*** for 10 min at 4°C. The cell-free extract was loaded on top of a three step isopycnic sucrose gradient containing fractions of 2 M sucrose, 1.5 M sucrose and 0.5 M sucrose and separated by ultracentrifugation at 100000 ***g*** for 1h at 4°C [28]. The enriched membrane fractions were collected and separated once more using the same method.

### Preparation of the periplasmic fraction from *P. aeruginosa*

For the preparation of the soluble periplasmic fraction, *P. aeruginosa* cells were resuspended in 50 mM Tris/HCl, pH 8, containing 20% (w/v) sucrose and 2 mg/ml polymyxin B sulphate and incubated over night at 4°C under soft movement. Alternatively, the *P. aeruginosa* cells were resuspended in 50 mM Tris/HCl, pH 7.5, containing 2 mg/ml polymyxin B sulphate and incubated for 1 h at 4°C. The periplasmic fraction was then isolated by centrifugation of the cell suspension for 30 min at 16100 ***g*** and 4°C.

### Preparation of immunoaffinity resin

The immunoaffinity resin consisting of a 1:1 mixture of protein A- and protein G-resin with covalently cross-linked NirS or NirN antibodies was prepared as described in the Supplementary Material (at http://www.bioscirep.org/bsr/033/bsr033e048add.htm).

### Immunoaffinity chromatography

For the immunoaffinity purification of native NirS or NirN, 2 g of anaerobically grown *P. aeruginosa* PAO1 cells were resuspended in 10 ml of lysis buffer and treated as described above for the co-immunoprecipitation. Then, 10 ml of cell-free extract were mixed with 5 ml of binding buffer and pre-cleared by the addition of 300 μl of protein A/protein G resin (1:1 slurry) for 15 min at 4°C with soft movement. The pre-cleared extract was separated from the resin by centrifugation for 2 min at 16100 ***g*** and 4°C. Afterwards, the pre-cleared extract was incubated for 2 h at 4°C and soft movement with 150 μl prepared immunoaffinity resin. The resin was collected in an empty column and washed first with 60 CV (column volume) TBS, 30 CV TBS-Tween (0.1%), 30 CV TBS containing 500 mM NaCl and 30 CV co-immunoprecipitation washing buffer. Finally, the loaded resin was incubated with 4 CV of elution buffer according to the GenScript resin manual for 15 min at 37°C and eluted by centrifugation in an empty Mini Bio-Spin column at 1000 ***g***. Afterwards, the resin was incubated for 5 min with 4 CV TBS at room temperature and eluted again by centrifugation. For the last elution step the material was incubated in 4 CV of 50 mM diethylamine, pH 11.5, with 0.5% sodium-deoxycholate for 15 min at 37°C [29] and the flow-through was collected by centrifugation. All collected fractions were neutralized by the addition of 1/10 (v/v) of 1 M Tris/HCl, pH 8, immediately after centrifugation. The elution fractions were pooled and concentrated in Millipore Spin concentrators (< 3 kDa pore size), heated with 6×SDS/PAGE sample buffer containing 2-mercaptoethanol for 10 min at 95°C and analysed by SDS/PAGE using Mini-PROTEAN any kD TGX Precast Gels (Bio-Rad Laboratories GmbH). The gels were stained with SYPRO® Ruby and visualized on a UV-screen. Stained protein bands were compared with the samples obtained from *P. aeruginosa* PAO1 WT and *P. aeruginosa* PAO1 RM488 or RM361.

### Nano HPLC-MS/MS-based protein identification

Protein digestion, peptide isolation and desalination were carried out according to Toyofuku *et al.* [30] with double concentrated NH_4_HCO_3_ buffer, 30 min of carbamidomethylation and only one step of formic acid extraction (5% (v/v) formic acid). Prior to analysis the samples were resuspended in 11 μl of 3% (v/v) acetonitrile/0.1% (v/v) formic acid. The separation of the peptide samples was performed using Ultimate 3000 RSLCnano-HPLC system (Dionex) as previously described [31]. MS and MS/MS data were acquired using an Orbitrap velos mass spectrometer (Thermo). Doubly and triply charged peptide-ions were automatically selected and fragmented with m/z-dependent collision energy settings. The raw data files were processed using the Mascot Deamon software (V2.32). Database searches were carried out with a local Mascot server (V2.2) in a *P. aeruginosa* PAO1 database (NCBI 2010-06-03) for the NirN samples using the given settings (enzyme: trypsin; maximum missed cleavages: 1; fixed modification: carbamidomethyl (Cys); and variable oxidation (Met); peptide mass tolerance: 20 ppm; MS/MS tolerance: 0.3 Da). The results of the NirS samples were obtained from the NCBI database (2011-06-11). Identification was regarded as valid with a significance value of *P*<0.05 that the observed match is a random event.

### UV–visible absorption spectroscopy

The UV–visible absorption spectra of the periplasmic protein fractions of *P. aeruginosa* cells were recorded using a V-650 spectrophotometer (Jasco).

## RESULTS AND DISCUSSION

In this study, we wanted to test the two hypotheses that the NirN protein is either required for the insertion of haem *d*_1_ into the cytochrome *cd*_1_ nitrite reductase NirS or involved in both the last step of haem *d*_1_ biosynthesis and the insertion of the cofactor into NirS. In either case, it is reasonable to assume that NirN and NirS will interact with each other, at least transiently. Therefore one major aim of this study was to investigate the potential *in vivo* interactions between NirS and NirN through co-immunoprecipitation of the two proteins from a *P. aeruginosa* PAO1 cell-free extract. For this purpose, we first determined the amounts of NirS and NirN produced by *P. aeruginosa* under anaerobic denitrifying growth conditions at different time points.

### *P. aeruginosa* produces lower amounts of NirN than NirS during anaerobic growth

**Figure 2:**
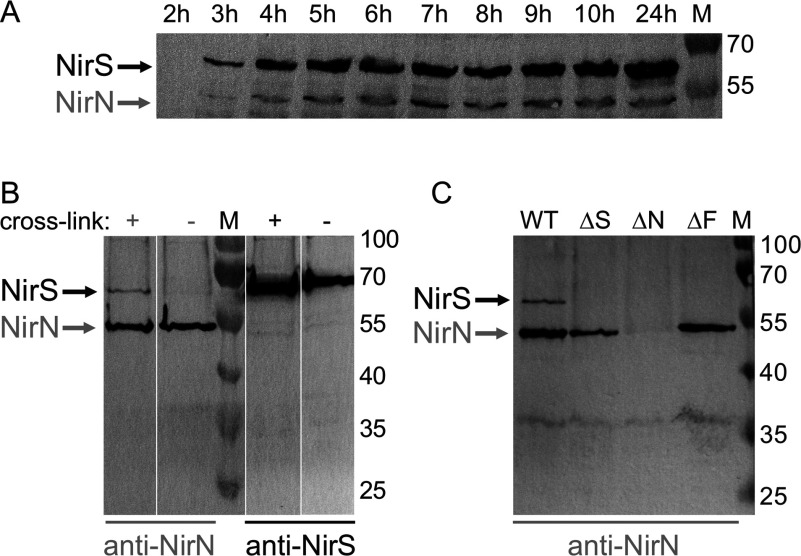
Interactions between NirS and NirN (**A**) Production of NirS and NirN in *P. aeruginosa*. *P. aeruginosa* PAO1 WT was grown anaerobically in the presence of nitrate. At indicated time points, samples were taken from the culture and the proteins were separated by SDS/PAGE. After Western blotting, NirS and NirN were visualized using NirS- and NirN-antibodies. (**B**) Co-immunoprecipitation of NirS and NirN. The co-immunoprecipitation of NirN and NirS from cell free extracts of *P. aeruginosa* PAO1 WT using NirN- and NirS-antibodies was performed as described in the Materials and Methods section. The experiments were conducted with (+) or without (−) *in vivo* protein cross-linking. After SDS/PAGE and Western blotting, NirS and NirN were visualized using NirS and NirN-antibodies. (**C**) Co-immunoprecipitation of NirS with NirN. The co-immunoprecipitation of NirS with NirN was performed using a NirN-antibody after *in vivo* cross-linking with cell free extracts of *P. aeruginosa* PAO1 WT, RM488 (ΔS), RM361 (ΔN) and RM301 (ΔF) strains. After SDS/PAGE and Western blotting, NirS and NirN were visualized using NirS- and NirN-antibodies. Lanes M, marker proteins with M_r_ values indicated. For all experiments equal amounts of cells (by weight) were used.

The analysis of the production of NirN and NirS in *P. aeruginosa* grown anaerobically in the presence of nitrate showed that the amounts of NirN remained almost constant over a time period of 20 h, whereas the amounts of NirS steadily increased (Figure 2A). Moreover, NirN was produced in significantly lower amounts than NirS. For the subsequent co-immunoprecipitation experiments, we always used *P. aeruginosa* cultures after 8 h of growth.

### NirS co-precipitates with NirN after *in vivo* protein cross-linking

The co-immunoprecipitation experiments were performed as described in the Materials and Methods section. In a first experiment, we tried to co-precipitate NirN and NirS without previous cross-linking of the proteins. However, under the conditions used no interaction between the two proteins was observed. Consequently, we repeated the same experiment after *in vivo* protein cross-linking with formaldehyde. Under these conditions, we found that NirS co-precipitated with NirN, which was pulled down with a NirN-antibody, providing evidence for an *in vivo* interaction between NirN and NirS for the first time (Figure 2B). The fact that this interaction was only observed after trapping the NirN–NirS complex by *in vivo* protein cross-linking indicates that the interaction between NirN and NirS is quite weak or occurs only transiently. However, such a weak and/or transient interaction is consistent with a role of NirN as a haem *d*_1_ insertion protein from which NirS dissociates as soon as the cofactor is delivered.

When NirS was pulled down with a NirS-antibody, no co-precipitated NirN was detected (Figure 2B). However, this observation can be explained by (a) the lower amounts of produced NirN relative to NirS and (b) the fact that the majority of the NirS proteins in the cell is expected to be in the haem *d*_1_-containing holo form. Assuming that NirN interacts with NirS only during the insertion of the haem *d*_1_, that is, with the apo or semi-apo (haem *c*-containing) NirS, which represents the minority of the total NirS population, the lack of detectable amounts of co-precipitated NirN is not surprising.

### The NirN–NirS interaction is dependent on the presence of NirF

In addition to the co-immunoprecipitation of NirS with NirN in the *P. aeruginosa* PAO1 WT strain, we also performed the same experiment with the *P. aeruginosa* PAO1 mutant strains RM488, RM361 and RM301, which carry the tetracycline resistance gene (*tet*) in place of the genes *nirS*, *nirN* and *nirF*, respectively [11]. It was shown previously that NirS is produced in the PAO1 strains RM361 and RM301 [11]. For the strain RM488 lacking the *nirS* gene, it was possible to pull down NirN using the NirN-antibody, but no NirS was visible, as expected (Figure 2C). When the same experiment was performed with the strain RM361 lacking *nirN* neither NirN nor NirS were detected. This result clearly showed that the co-precipitation of NirS with NirN in the WT strain was not due to unspecific binding of NirS to the protein A sepharose material. Interestingly, for the strain RM301 lacking the *nirF* gene we did also not detect any co-precipitated NirS. This observation indicated that the interaction between NirN and NirS was dependent on the presence of NirF.

### NirS co-purifies with NirF after *in vivo* protein cross-linking

**Figure 3:**
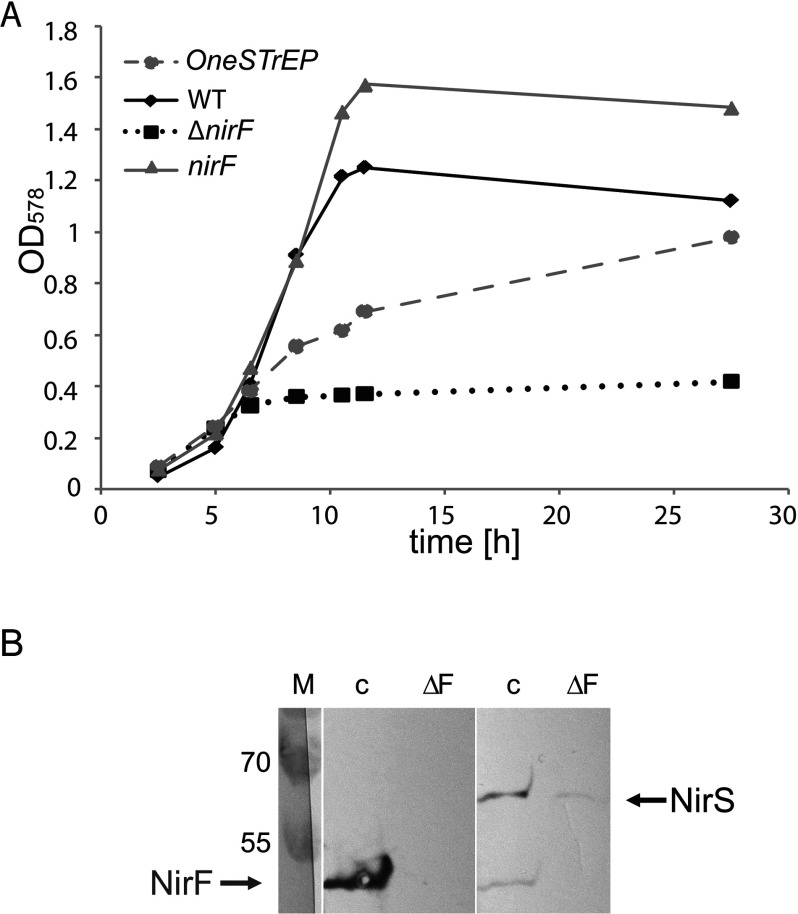
Interactions between NirS and NirF (**A**) Growth curves of *P. aeruginosa* PAO1 WT and *P. aeruginosa* PAO1 RM301. *P. aeruginosa* was grown under anaerobic conditions in the presence of nitrate as described in the Materials and Methods section. Strain PAO1 RM301 (■) showed strongly impaired growth under these conditions compared with the WT strain (◆). The growth of strain PAO1 RM301 was partially restored by plasmid pUCP20T*nirFOneSTrEP* (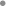) and fully restored by plasmid pUCP20T*nirF* (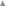). (**B**) Affinity co-purification of NirS with NirF-STrEP. STrEP-tagged NirF was produced in the *P. aeruginosa* PAO1 RM301 strain carrying pUCP20T*nirFOneSTrEP* and was purified using Strep-Tactin sepharose after *in vivo* protein cross-linking. The eluted proteins were separated by SDS/PAGE and, after Western blotting, visualized using Strep-Tactin AP conjugate (lane c, left hand side) or NirS-antibodies (lane c, right hand side). The same experiment was performed with *P. aeruginosa* PAO1 RM301 that did not produce NirF–STrEP (lanes ΔF). For the preparation of the figure the lanes on the left and right hand sides were put together from two differently stained blots of the same sample. The STrEP-tagged NirF runs higher in SDS/PAGE gels than native NirF due to the STrEP-tag. Lane M, marker proteins with M_r_ values indicated. For all experiments equal amounts of cells (by weight) were used.

One potential mechanism explaining the involvement of NirF in the interaction between NirN and NirS was the possibility that NirF also directly participates in the transient interactions between these two proteins. In order to test this hypothesis, we performed co-purification experiments after *in vivo* protein cross-linking. For the co-purification of interaction partners with NirF, the STrEP-tagged (C-terminal) version of NirF was produced in the *P. aeruginosa* PAO1 strain RM301 lacking the native NirF. As shown in Figure 3A, the STrEP-tagged NirF partially rescued the growth phenotype of strain RM301 indicating that NirF retained its function despite the C-terminal STrEP-tag and that the production of an active NirS was partially restored. After 8 h of growth and *in vivo* protein cross-linking with formaldehyde, a cell-free extract was prepared and the NirF–STrEP together with its cross-linked interaction partners was purified using Strep-Tactin sepharose as described in the Materials and Methods section. After SDS/PAGE and Western blotting, the purified NirF–STrEP was visualized using a Strep-Tactin AP conjugate. Using a NirS-antibody, we also detected NirS that was co-purified with NirF (Figure 3B). In contrast, NirN was not co-purified with NirF in detectable amounts (results not shown). When the same experiment was performed with a cell-free extract of strain RM301, that did not produce NirF–STrEP, only traces of NirS were retained on the Strep-Tactin sepharose showing that NirS was indeed co-purified with NirF in the former experiment. The results of these experiments suggested a direct interaction between NirS and NirF. Although we did not detect NirN as an interaction partner of NirF in this particular experiment, it should not be ruled out that NirN and NirF might also directly interact with each other. In fact, the dependence of the NirN–NirS interaction on the presence of NirF (described above) could be nicely explained if NirN would make contacts with both NirS and NirF. Possibly, we were not able to detect NirN as a direct interaction partner of NirF through affinity co-purification due to the C-terminal STrEP-tag. As mentioned above, the NirF–STrEP fusion protein only partially rescued the growth phenotype of strain RM301 in contrast to the untagged NirF protein, which fully restored the anaerobic growth of the Δ*nirF* mutant (Figure 3A). These differences in the ability to complement the Δ*nirF* mutant strain might be due to the partial disturbance of protein interactions between NirF–STrEP and its periplasmic partner proteins. As a consequence, we also tried to use an N-terminally modified NirF for the same experiments. Unfortunately, the modification of the N-terminus of NirF always resulted in the complete loss of *in vivo* activity (not shown) and, therefore, these constructs could not be used for the affinity co-purification of NirF with its interaction partners.

### NirS, NirN and NirF co-purify after *in vivo* protein cross-linking

**Figure 4:**
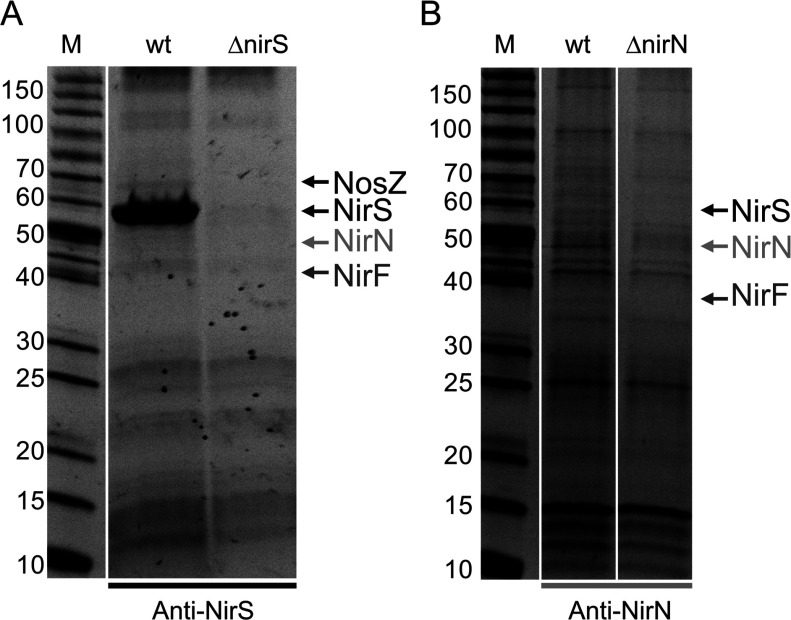
Immunoaffinity chromatography of NirS (A) and NirN (B) Native NirS and NirN were purified from cell free extracts of *P. aeruginosa* PAO1 WT after *in vivo* protein cross-linking using anti-NirS (**A**) and anti-NirN (**B**) immunoaffinity chromatography columns. The same experiments were done with cell free extracts of *P. aeruginosa* PAO1 strains RM488 (Δ*nirS*) and RM361 (Δ*nirN*) as a control. The protein content of the elution fractions was analysed by SDS/PAGE and the proteins were visualized with SYPRO® Ruby under UV light. The indicated protein bands of the WT samples were cut from the polyacrylamide gel and analysed by MS. Some of the detected proteins are indicated. For a complete list of detected proteins see also Supplementary Tables S2 and S3. Lanes M, marker proteins with M_r_ values indicated. For all experiments equal amounts of cells (by weight) were used.

In order to further study the interaction network between NirS, NirN and NirF, we performed immunoaffinity chromatography of native NirS and NirN after *in vivo* protein cross-linking with subsequent identification of the co-purified proteins by MS. For this purpose, we generated anti-NirS and anti-NirN immunoaffinity chromatography columns by coupling the respective antibodies covalently to a mixture of protein A and protein G sepharose resin as described in the Supplementary Materials and Methods (at http://www.bioscirep.org/bsr/033/bsr033e048add.htm). When NirS was purified from a cell-free extract of *P. aeruginosa* PAO1 WT cells using the anti-NirS immunoaffinity column many other proteins co-eluted together with NirS according to SDS/PAGE analysis (Figure 4A). In order to determine the non-NirS-bound protein background, the same experiment was performed with a cell-free extract of the *P. aeruginosa* PAO1 RM488 strain lacking NirS as a control (Figure 4A). Subsequently, only those protein bands were analysed by MS that were visible in the NirS-co-elution fraction and were not present in the control experiment. As shown in Figure 4 and listed in Supplementary Tables S2 and S3 (at http://www.bioscirep.org/bsr/033/bsr033e048add.htm), we detected NirN and NirF that were co-purified with NirS. Thus, the results obtained by co-immunoprecipitation and affinity co-purification described above were confirmed. Moreover, we also identified co-purified NosZ by MS (Figure 4A and Supplementary Table S2). Analogously, when NirN was purified using an anti-NirN immunoaffinity column we detected co-purified NirS and NirF (Figure 4B). Again, only those protein bands were analysed by MS that were visible in the NirN-co-elution fraction and not in the elution fraction of the corresponding control experiment using the *P. aeruginosa* PAO1 RM361 strain lacking NirN (Figure 4B).

The results of these immunoaffinity chromatography experiments together with the results of the co-immunoprecipitation and the affinity co-purification clearly suggest that all three proteins NirS, NirN and NirF interact with each other *in vivo* and form a weak and transient ternary complex during the maturation (i.e. haem *d*_1_ insertion) of NirS. Of course, these experiments do not tell us anything about the stoichiometry of the proteins within the complex or about the arrangement of the three proteins relative to each other.

Furthermore, NosZ was also co-purified on the anti-NirS affinity column suggesting an interaction between NosZ and NirS. NosZ catalyses the last step during denitrification, namely the reduction of N_2_O (nitrous oxide) to dinitrogen [1]. Presumably, the last three denitrification enzymes, i.e. NirS (nitrite reductase), NorBC (NO reductase) and NosZ (N_2_O reductase), form a weak complex *in vivo* and, thus, we found NosZ as an interaction partner of NirS in our immunoaffinity chromatography experiments. NorBC was not found to be co-purified with NirS in significant amounts since this protein is bound within the membrane [32,33]. Although the buffers used for immunoaffinity chromatography contained detergents, the short time frame between cell disruption and centrifugation probably prevented the solubilization of integral membrane proteins. On the other hand, NirF that was predicted to be a membrane-anchored lipoprotein [18] was detected in the immunoaffinity chromatography experiments and was present in the soluble protein fraction (also prepared with detergent containing buffer) used for the affinity co-purification on Strep-Tactin sepharose. Apparently, the buffer conditions and sample handling were suitable in order to solubilize the proposed lipoprotein NirF. However, since NirF from *P. aeruginosa* was so far only bioinformatically predicted to be a lipoprotein, we wanted to verify this prediction experimentally.

### NirF from *P. aeruginosa* is a membrane attached lipoprotein

**Figure 5:**
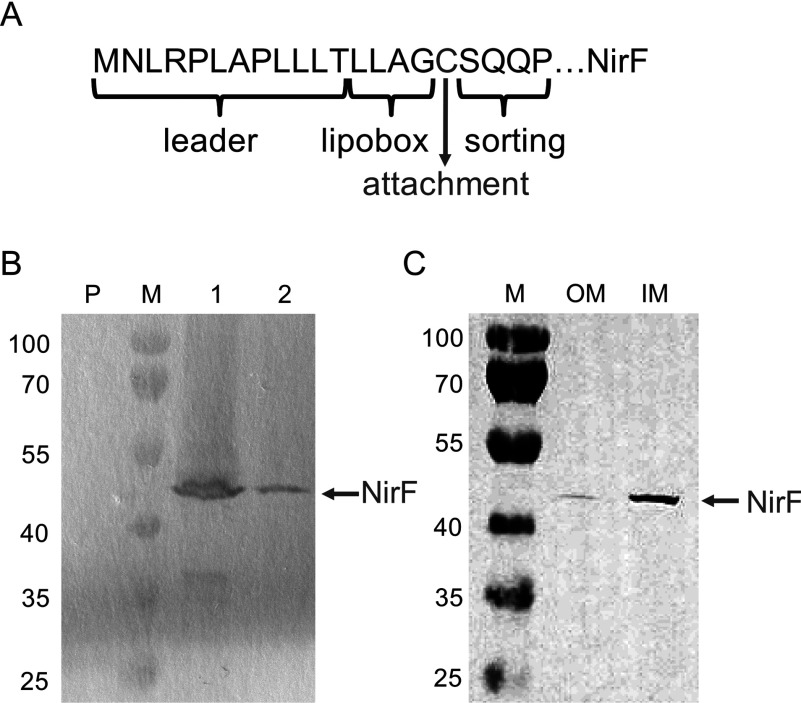
Localization of NirF (**A**) N-terminal amino acid sequence of NirF from *P. aeruginosa*. The N-terminal NirF sequence contains features characteristic for a periplasmic lipoprotein such as a leader sequence for the export to the periplasm, a lipobox, a cysteine residue as the attachment site for the lipid anchor and a sorting sequence that determines the localization of the protein in the IM (inner membrane) or OM (outer membrane). (**B**) Localization of NirF in the membrane fraction. STrEP-tagged NirF was produced in *P. aeruginosa* PAO1 RM301 and the periplasmic and membrane fractions were prepared. After SDS/PAGE and Western blotting, the NirF–STrEP was visualized using Strep-Tactin AP conjugate. Lane P represents the periplasmic fraction, lane 1 the membrane fraction and lane 2 the solubilized membrane proteins. (**C**) Localization of NirF in the IM. STrEP-tagged NirF was produced in *P. aeruginosa* PAO1 RM301 and the IM and OM fractions were prepared as described in the Materials and Methods section. After SDS/PAGE and Western blotting, the NirF–STrEP was visualized using Strep-Tactin AP conjugate. Lanes M, marker proteins with M_r_ values indicated.

Originally, NirF from *P. aeruginosa* was annotated as a cytoplasmic protein in the databases. However, in a recent study, in which the localization of all predicted lipoproteins in *P. aeruginosa* was reanalysed, NirF was listed as a proposed lipoprotein potentially attached to the periplasmic side of the inner membrane *via* a lipid anchor [18]. In contrast, NirF from *P. pantotrophus* was reported to be a soluble, periplasmic protein [17]. However, in contrast to the *P. pantotrophus* enzyme, the N-terminal amino acid sequence of *P. aeruginosa* NirF indeed contains sequence motifs such as a lipobox, a cysteine residue as lipid attachment site and a sorting sequence, which classify this protein as a lipoprotein (Figure 5A). In order to experimentally verify the bioinformatic prediction that NirF from *P. aeruginosa* is a membrane attached lipoprotein, we produced the STrEP-tagged (C-terminal) version of NirF in *P. aeruginosa* PAO1 RM301 lacking the native NirF as described above for the affinity co-purification experiments. After 8 h of growth the cells were harvested and the periplasmic and membrane fractions were prepared. After SDS/PAGE and Western blotting the STrEP-tagged NirF was detected exclusively in the membrane fraction and not in the soluble periplasmic fraction showing that NirF is indeed a membrane attached protein in *P. aeruginosa* (Figure 5B). Next, we wanted to test whether NirF is attached to the inner or the outer membrane. For this purpose, STrEP-tagged NirF was produced in *P. aeruginosa* RM301 and the membrane fraction was prepared as before. In addition, the inner and outer membrane fractions were separated by ultracentrifugation using a three-step sucrose gradient as described in the Materials and Methods section. After SDS/PAGE and Western blotting we detected the STrEP-tagged NirF mainly in the inner membrane fraction (Figure 5C). Only minor amounts of NirF–STrEP were detected in the outer membrane fraction probably due to incomplete separation of the two fractions. Thus, we clearly showed that NirF from *P. aeruginosa* is attached to the inner membrane most likely *via* a lipid anchor.

The experiments described so far showed that the three proteins NirF, NirN and NirS form a weak, membrane-associated complex *in vivo*. In this complex, NirF was supposed to catalyse the last step of haem *d*_1_ biosynthesis, that is, the formation of the double bond of the acrylate side chain of haem *d*_1_ [16], and NirS is, of course, the target protein into which the haem *d*_1_ cofactor is inserted. However, the role of NirN within the ternary NirF–NirN–NirS complex was still unclear. Possible functions of NirN could be (a) that of a haem *d*_1_ insertion protein required for the correct incorporation of haem *d*_1_ into NirS, (b) that of a haem *d*_1_ biosynthesis protein catalysing the dehydrogenation of dihydrohaem *d*_1_ together with NirF or (c) that of both, a haem *d*_1_ biosynthesis and insertion protein. In order to gain more insights into the physiological role of NirN, we decided to investigate whether the knock out of the *nirN* gene in *P. aeruginosa* resulted in alterations of the haem *d*_1_ content of NirS. Previously, it was observed that the *nirN* knock out in *P. aeruginosa* resulted in less NirS activity *in vivo* and *in vitro* [11,19]. However, it was not determined whether the lower level of NirS activity was due to differences in the cofactor content of NirS depending on whether the protein was produced in the WT strain or the *P. aeruginosa* PAO1 RM361 strain lacking *nirN*.

### Knock out of the *nirN* gene alters the cofactor content of NirS in *P. aeruginosa*

**Figure 6:**
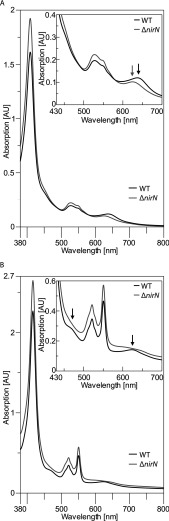
UV–visible absorption spectra of the periplasmic protein fraction of *P. aeruginosa* The periplasmic protein fractions of anaerobically grown *P. aeruginosa* PAO1 WT (black lines) and *P. aeruginosa* PAO1 RM361 (Δ*nirN*, grey lines) were prepared and UV–visible absorption spectra were recorded for the as-isolated form (**A**) and the dithionite reduced form (**B**). The differences between the absorption spectra of the WT and the Δ*nirN* sample are highlighted by arrows.

In order to study the effects of the *nirN* knock out, we prepared the periplasmic protein fractions from anaerobically grown cells of *P. aeruginosa* PAO1 WT and the *P. aeruginosa* PAO1 mutant strain RM361 [11], which carries the tetracycline resistance gene (*tet*) in place of *nirN*, and recorded UV–visible absorption spectra of these samples (Figure 6). In the as-isolated (oxidized) form, the UV–visible absorption spectra of the WT sample and the Δ*nirN* sample displayed identical features at 409, 527 and 549 nm representing the mixture of periplasmic *c*-type cytochromes present in both samples. In addition, the sample prepared from the WT cells exhibited an absorption peak at 638 nm, whereas the sample prepared from the Δ*nirN* mutant showed a less intense absorption peak at 624 nm (Figure 6A). The absorption peak at 638 nm observed for the WT sample represents the characteristic absorption feature of the oxidized form of haem *d*_1_-containing NirS [34]. In contrast, the absorption peak at 624 nm observed for the sample prepared from the Δ*nirN* mutant is not in line with the presence of haem *d*_1_-containing holo NirS in the oxidized form. We also recorded UV–visible absorption spectra of the same samples after the addition of sodium dithionite in order to reduce all the periplasmic cytochromes including NirS. The spectra of the reduced forms of both samples exhibited the typical absorption features of reduced *c*-type cytochromes at 417, 521 and 551 nm (Figure 6B). In addition, the spectrum of the WT sample exhibited an absorption peak at 623 nm and a shoulder at around 468 nm, which are characteristic features of dithionite-reduced haem *d*_1_ bound to NirS according to the literature [34–37]. In contrast, instead of a distinct peak at 623 nm, the spectrum of the Δ*nirN* sample exhibited a very broad absorption feature between 580 and 670 nm, and there was no shoulder at 468 nm.

Based on these results, we conclude that in the absence of NirN the formation of the conventional haem *d*_1_-containing holo NirS is impaired. Instead, a so far uncharacterized form of NirS is made, which is catalytically active in the Δ*nirN* mutant, but displays absorption features distinct from those of the conventional haem *d*_1_-containing holo NirS. One possible explanation for our results could be that NirN is required for the last step of haem *d*_1_ biosynthesis, namely the formation of the double bond of the acrylate side chain (Figure 1B). Possibly, both proteins NirF and NirN might be required for this reaction. If this hypothesis was true, haem *d*_1_ biosynthesis would stop at the stage of the haem *d*_1_ precursor, dihydrohaem *d*_1_, which might bind to semi-apo NirS *in vivo* yielding a semi-functional NirS in the Δ*nirN* mutant. Indeed, it was shown previously *in vitro* that NirS carrying dihydrohaem *d*_1_ instead of the true haem *d*_1_ was catalytically active [20]. Moreover, the fact that the characteristic haem *d*_1_ absorption band around 640 nm observed in the WT sample is shifted to 624 nm in the Δ*nirN* sample (Figure 6A) can indeed be interpreted in favour of the missing acrylate double bond (hypsochromic shift) and, thus, the presence of the haem *d*_1_ precursor in the Δ*nirN* mutant. Previous spectroscopic studies on isolated haem *d*_1_ had also shown that the hydrogenation of the acrylic double bond leads to a shift of the long wavelength absorption band of haem *d*_1_ to a shorter wavelength [38]. Therefore we postulate that the catalytically active NirS present in the cells of the *P. aeruginosa* PAO1 Δ*nirN* mutant carries the haem *d*_1_ precursor dihydrohaem d_1_.

In order to further substantiate this proposal, we also purified the native NirS produced in the *P. aeruginosa* PAO1 Δ*nirN* mutant to obtain the protein with the potentially bound dihydrohaem *d*_1_. Unfortunately, the purified NirS was in the semi-apo form and did not contain any non-covalently bound cofactor as judged by UV–visible absorption spectroscopy (results not shown). However, this result additionally indicated that the conventional haem *d*_1_ was not produced and not incorporated into NirS in the Δ*nirN* mutant strain, since haem *d*_1_ is tightly bound in the protein and usually not lost during the purification of NirS.

### Potential model for the biosynthesis of haem *d*_1_ and its incorporation into NirS

Based on the results obtained in this study, we propose a potential model for the biosynthesis of haem *d*_1_ and its insertion into the cytochrome *cd*_1_ nitrite reductase NirS in *P. aeruginosa*. In this model, the first steps of haem *d*_1_ biosynthesis up to the stage of dihydrohaem *d*_1_ take place in the bacterial cytoplasm. Dihydrohaem *d*_1_ is then transported across the inner membrane *via* an as yet unknown mechanism. Possibly, a specific dihydrohaem *d*_1_ transporter might be required for this process. The membrane-anchored lipoprotein NirF that potentially interacts with the transporter then takes up the dihydrohaem *d*_1_ and potentially catalyses the dehydrogenation of one of the propionate side chains of the haem *d*_1_ precursor to yield the acrylate side chain of haem *d*_1_. Based on our results we propose that NirN is also required for this last haem *d*_1_ biosynthesis step. Finally, the fully synthesized haem *d*_1_ is then passed on to NirS yielding the holo enzyme. All three proteins NirF, NirN and semi-apo NirS interact with each other during the maturation of NirS after which the holo enzyme dissociates from the complex.

## Online data

Supplementary data
