# Supplementary material for: Maturation of the cytochrome cd1 nitrite reductase NirS from Pseudomonas aeruginosa requires transient interactions between the three proteins NirS, NirN and NirF
Source: Biosci Rep. 2013 Jun 27;33(3):e00048. doi: 10.1042/BSR20130043 (PMC3694632; doi:10.1042/BSR20130043)
Supplement: Supplementary data [file bsr033e048add.pdf]

Biosci. Rep. (2013) / **33** / art:e00048 / doi 10.1042/BSR20130043

OPEN ACCESS

Original Paper

Bioscience Reports

www.bioscirep.org

Bioscience Reports

SUPPLEMENTARY DATA

Maturation of the cytochrome cd_1 nitrite reductase NirS from *Pseudomonas aeruginosa* requires transient interactions between the three proteins NirS, NirN and NirF

Tristan NICKE*, Tobias SCHNITZER*, Karin MÜNCH*, Julia ADAMCZACK*, Kristin HAUFSCHILDT*, Sabine BUCHMEIER†, Martin KUCKLICK*, Undine FELGENTRÄGER‡, Lothar JÄNSCH‡, Katharina RIEDEL§ and Gunhild LAYER*[1]

*Institute of Microbiology, Technische Universität Braunschweig, Spielmannstr. 7, 38106 Braunschweig, Germany, †Institute of Physical and Theoretical Chemistry, Technische Universität Braunschweig, Hans-Sommer-Street 10, 38106 Braunschweig, Germany, ‡Department of Cellular Proteome Research, Helmholtz Centre for Infection Research, Inhoffenstr. 7, 38124 Braunschweig, Germany, and §Institute of Microbiology, Ernst-Moritz-Arndt University of Greifswald, Friedrich-Ludwig-Jahn-Street 15, 17489 Greifswald, Germany

MATERIALS AND METHODS

In vivo protein cross-linking

The *in vivo* protein cross-linking was performed as described previously with minor modifications. *P. aeruginosa* PAO1 strains were grown for 8 h at 37 °C under anaerobic conditions in LB (Luria–Bertani) medium supplemented with 50 mM $NaNO_3$. The cultures were then supplemented with 0.125 % of formaldehyde solution (37 %) and incubated for 20 min at 37 °C on an orbital shaker at 160 rpm. Cross-linking was stopped by addition of 125 mM (final concentration) glycine solution and incubation for 5 min at 37 °C and 160 rpm.

Western blotting

For the detection of NirS, NirN or STrEP-tagged NirF variants, samples were supplemented with SDS-sample buffer containing 2-mercaptoethanol, heated at 95 °C for 10 min and the proteins were separated on 12 % denaturating polyacrylamide gels. Afterwards, the proteins were blotted onto polyvinylidene fluoride membranes. Staining of the proteins was performed with the monoclonal antibodies 1A11 (αNirS) and 2C11 (αNirN) and alkaline phosphatase attached to a secondary antibody. NirF-OneSTrEP was directly probed with Strep-Tactin AP conjugate after masking biotinylated proteins with avidin.

Preparation of immuno-affinity resin

Protein A- and protein G-resin was mixed in a 1:1 ratio to give a 1 ml column volume and equilibrated with binding buffer according to the manufacturer's manual (GenScript). 1 mg of monoclonal antibodies (αNirS-mix) or polyclonal antibodies (αNirN) were bound to the column and washed with 50 column volumes (CV) of binding buffer at a flow rate of 1.3 ml/min. Afterwards, the immuno-affinity resin was stored in binding buffer supplemented with 0.02 % of NaN_3 or directly washed with 30 CV of 0.2 M triethanolamine, pH 8.2. The cross-linking of the antibodies with the immuno-affinity resin was performed with 20 CV of 70 mM dimethyl pimelimidate in 0.2 M triethanolamine, pH 8.2, for 45 min at room temperature. The resin was then collected by soft centrifugation and separation from the supernatant. The cross-linking was stopped by resuspending the resin in 25 CV of 70 mM ethanolamine, pH 8.2, and incubation for 5 min at room temperature. The resin was washed with 50 CV of binding buffer and stored for a short time in binding buffer containing 0.02 % NaN_3.

[1] To whom any correspondence should be addressed (email g.layer@tu-braunschweig.de).

© 2013 The Author(s) This is an Open Access article distributed under the terms of the Creative Commons Attribution Licence (CC-BY) (http://creativecommons.org/licenses/by/3.0/) which permits unrestricted use, distribution and reproduction in any medium, provided the original work is properly cited.

Table S1 Oligonucleotides and primers
Restriction sites are underlined

Number	Sequence (5′→3′)	Use
1	GACCATGGGTAAGGACGACATGAAAG	Amplification of *nir*S fw. without leader sequence (*Nco*I)
2	GTAAGCTTCAGTACACGTGCTGGG	Amplification of *nirS* rev. (*Hind*III)
3	CATGCCATGGGCGAAGCGCCG	Amplification of *nirN* fw. without leader sequence (*Nco*I)
4	CGGGATCCTCAGTGCGAGGTTC	Amplification of *nirN* rev. (*Bam*HI)
5	CATCGGATCCAGGAGAGATCGCC	Amplification of *nirF* fw. with *rbs* and leadersequence (*Bam*HI) and *nirFOneSTrEP* fw. (*Bam*HI)
6	CATCACTAGTGAGTCCGATGTGCTGGG	Amplification of *nirF* rev. without stop codon (*Spe*I)
7	CATCGCATGCTCACTACTTCTCGAACTG	Amplification of *nirFOneSTrEP* rev. (*Sph*I)
8	GTGCGGCCGCAAGCTTCTAGAGTCCGATGTGCTGG	QuikChange *nirF* fw. incorporate stop codon
9	CCAGCACATCGGACTCTAGAAGCTTGCGGCCGCAC	QuikChange nirF rev. incorporate stop codon
10	GCCGCCGGAGCCGCCCTACTTCTCGAACTGGGG	QuikChange StrepII fw. shorten STrEPOne Tag
11	CCCCAGTTCGAGAAGTAGGGCGGCTCCGGCGGC	QuikChange StrepII rev. shorten STrEPOne Tag

© 2013 The Author(s) This is an Open Access article distributed under the terms of the Creative Commons Attribution Licence (CC-BY) (http://creativecommons.org/licenses/by/3.0/) which permits unrestricted use, distribution and reproduction in any medium, provided the original work is properly cited.

Table S2 Periplasmic (P), membrane proteins (IM/OM) and outer membrane vesicle proteins (OMV) from *P. aeruginosa* PAO1
Corresponds to Figure 4(A) of the main text. Locations according to www.pseudomonas.com or SignalP3.0 Server. Trypsin-digested. Peptide Mass Tolerance, ±20 p.p.m. Fragment Mass Tolerance, ±0.3 Da. Significance Threshold $P < 0.05$. First 10 Results or Score above 45.

(a) Band, NosZ; band mass value, about 70 kDa

gi	Gene	Protein	Mass	Score	Sequences	Location
15595716	PA0519	NirS	62614	536	19	P
15598588	PA3392	NosZ	70615	150	7	P
15597002	PA1805	PpiD	68699	148	7	IM
15599791	PA4595	ABC-Transporter ATP Binding	61283	106	4	P
15597007	PA1810	ABC-Transporter component	70429	96	4	P
15599939	PA4745	NusA	54626	95	4	P
15596780	PA1583	SdhA	63492	76	2	P
15595785	PA0588	Hypothetical	73676	76	3	OMVesicle
15595715	PA0518	NirM	10960	66	1	P
15600230	PA5037	Hypothetical	57412	61	2	IM

(b) Band, NirS; band mass value about 60 kDa.

gi	Gene	Protein	Mass	Score	Sequences	Location
15595716	PA0519	NirS	62614	7843	35	P
15599581	PA4358	GroEL	57050	685	15	P/OMVes.
15599698	PA4502	ABC-Transporter component	58575	132	4	P
15599687	PA4491	Hypothetical	64175	78	3	OMVesicle
15596997	PA1800	Tig	48552	66	2	P
15596580	PA1383	Hypothetical	63104	65	2	P
15597001	PA1804	HupB	9081	57	2	OMVesicle
15598994	PA3799	EngA	54972	57	3	IM
15598588	PA3392	NosZ	70615	54	2	P
15600244	PA5051	ArgS	65158	53	2	P

(c) Band, NirN; band mass value, about 50 kDa

gi	Gene	Protein	Mass	Score	Sequences	Location
15595716	PA0519	NirS	62614	411	10	P
15596997	PA1800	Tig	48552	224	9	P
15597001	PA1804	HupB	9081	218	4	OMVescile
15598941	PA3746	Ffh	49328	161	3	IM
15595706	PA0509	NirN	53945	99	2	P
15600432	PA5239	Rho	47154	93	4	P
15596352	PA1155	NrdB	47356	76	3	P
15599026	PA3831	PepA	52299	61	2	OMVescile
15596202	PA1005	Hypothetical	52961	60	2	P
15598197	PA3001	Glycerald.-3-phosph. dehyd.	50080	60	2	P

(d) Band, NirF; band mass value, about 40 kDa

gi	Gene	Protein	Mass	Score	Sequences	Location
15595716	PA0519	NirS	62614	863	17	P
15595791	PA0594	SurA	48443	220	9	P
15596169	PA0972	TolB	47722	219	7	P
15600135	PA4942	HflK	44018	203	4	OMVesicle
15596271	PA1074	BraC	39744	192	5	P
15598813	PA3617	RecA	36856	185	4	C/P/OMV
15600451	PA5258	Hypothetical	40767	170	6	IM
15595713	PA0516	NirF	43311	170	6	P
15596785	PA1588	SucC	41517	111	4	P
15598355	PA3159	WbpA	48125	106	3	P/OMV

© 2013 The Author(s) This is an Open Access article distributed under the terms of the Creative Commons Attribution Licence (CC-BY) (http://creativecommons.org/licenses/by/3.0/) which permits unrestricted use, distribution and reproduction in any medium, provided the original work is properly cited.

Table S3 Periplasmic (P), membrane proteins (IM/OM) and outer membrane vesicle proteins (OMV) from *P. aeruginosa* PAO1
Corresponds to Figure 4(B) of the main text. Locations according to www.pseudomonas.com or SignalP3.0 Server. Trypsin-digested. Peptide Mass Tolerance, ±20 p.p.m. Fragment Mass Tolerance, ±0.3 Da. Significance Threshold $P < 0.05$. First 10 Results or Score above 45.

(a) Band, NirS; band mass value, about 60 kDa

gi	Gene	Protein	Mass	Score	Sequences	Location
15599581	PA4358	GroEL	57050	731	15	P/OMVes.
15595716	PA0519	NirS	62614	522	12	P
15599525	PA4329	PykA	52220	339	8	P
15598172	PA2976	Rne	117395	276	8	OMVes.
15599069	PA3874	NarH	58068	264	7	OMVes.
15600271	PA5078	Glucon biosynth. Protein G	59439	212	7	P
15596997	PA1800	Tig	48552	151	4	P
15600749	PA5556	AtpA	55359	136	4	P
15597035	PA1838	CysI	62094	122	3	P
15598358	PA3162	RpsA	61832	111	2	P/OMVes.

(b) Band, NirN; band mass value, about 50 kDa

gi	Gene	Protein	Mass	Score	Sequences	Location
15600432	PA5239	Rho	47040	525	14	P/IM
15595706	PA0509	NirN	53945	383	9	P
15596997	PA1800	Tig	48552	352	10	P
15596352	PA1155	NrdB	47356	213	4	P
15599581	PA4358	GroEL	57050	201	4	P/OMVes.
15598197	PA3001	Glyceraldehy.-3-phosph DH	50080	199	5	P/OMVes.
15596291	PA1094	FliD	49420	196	6	P/OMVes.
15600747	PA5554	AtpD	49469	188	5	P/OMVes.
15598172	PA2976	Rne	117395	170	5	OMVes.
15600189	PA4669	RfaE	50318	162	5	P

(c) Band, NirF; band mass value, about 38 kDa

gi	Gene	Protein	Mass	Score	Sequences	Location
15596534	PA1337	AnsB	38620	195	7	P
15600425	PA5232	Hypothetical	38549	135	3	OMVesicle
15598363	PA3167	SerC	39924	120	2	P
15597638	PA2442	GcvT2	39857	85	3	P
15595497	PA0300	SpuD	40604	83	2	P
15599581	PA4358	GroEL	57050	72	2	P/OMVes.
15597936	PA2740	PheS	38039	70	2	P
15600035	PA4842	Hypothetical	39964	65	1	OMVesicle
15598734	PA3538	ABC-Transporter component	39166	61	2	IM
15595713	PA0516	NirF	43311	60	2	P

Received 18 April 2013/8 May 2013; accepted 17 May 2013

Published as Immediate Publication 19 May 2013, doi 10.1042/BSR20130043

© 2013 The Author(s) This is an Open Access article distributed under the terms of the Creative Commons Attribution Licence (CC-BY) (http://creativecommons.org/licenses/by/3.0/) which permits unrestricted use, distribution and reproduction in any medium, provided the original work is properly cited.
